# Knowledge, attitudes and perceptions of autism spectrum disorder: a general public survey in Greece

**DOI:** 10.1192/j.eurpsy.2023.1023

**Published:** 2023-07-19

**Authors:** R. Kouznetsov, E. Jelastopulu

**Affiliations:** Department of Public Health, Medical School University of Patras, Patras, Greece

## Abstract

**Introduction:**

According to the World Health Organisation (March 2022), it is estimated that one in 100 children worldwide has autism spectrum disorder (ASD). People with ASD very often face stigma, discrimination, and violations of their human rights. The aim of this study was to examine the knowledge and the attitudes of the general population regarding autism spectrum disorder as well as to raise awareness and promote appropriate behaviors towards people with ASD.

**Objectives:**

The purpose of this study was to investigate the knowledge, attitudes, and the behaviors of the general population towards people with autism spectrum disorder in Greece. As far as is known, this is the first study carried out in Greece on this research topic.

**Methods:**

A cross-sectional study was conducted online via social media and completed by 642 participants with various characteristics. The questionnaires were anonymous, their completion was voluntary and included the “Societal Attitudes Towards Autism” (SATA) scale. Linguistic validation and cultural adaptation of the SATA scale into the Greek language was based on the World Health Organization specifications. Data analysis was performed using the Statistical Package for the Social Sciences.

**Results:**

A total of 642 people participated in the survey, 81.2% women and 18.8% men. The majority had a very good knowledge of autism (mean score 9.1 out of 13). However, women achieved significantly higher scores than men (p = 0.003). Respondents showed moderate to positive attitudes towards people with ASD (mean score 57.9 out of 80). The knowledge score is associated with the level of education, the place of residence, the existence of a person with autism in their friend or family environment, their income and occupation. The highest scores were observed in psychiatrists, following by teachers and other mental health professionals. Notably, 220 people believed autism to be caused by vaccination.

**Conclusions:**

Educational and public health interventions should be organized in the general population in Greece to discard childhood vaccination as a causative factor of autistic spectrum disorder. There is significant need to educate the public in acquiring knowledge about autistic spectrum disorder.

**Disclosure of Interest:**

None Declared

